# Age- and sex-specific reference values for CT-based low skeletal muscle quantity and quality in healthy living kidney donors

**DOI:** 10.3389/fphys.2025.1566463

**Published:** 2025-04-25

**Authors:** David Martin, Paola Dolce, Martin Hübner, Tobias Zingg, David Fuks, Jean-Pierre Venetz, Damien Maier, Maurice Matter, Fabio Becce

**Affiliations:** ^1^ Department of Visceral Surgery, Lausanne University Hospital (CHUV), University of Lausanne (UNIL), Lausanne, Switzerland; ^2^ Department of Transplantation, Lausanne University Hospital (CHUV), University of Lausanne (UNIL), Lausanne, Switzerland; ^3^ Department of Diagnostic and Interventional Radiology, Lausanne University Hospital (CHUV), University of Lausanne (UNIL), Lausanne, Switzerland

**Keywords:** living kidney donor, computed tomography, muscle mass, muscle quality, myosteatosis

## Abstract

**Background:**

Computed tomography (CT) imaging is a useful tool for assessing skeletal muscle mass and quality. The present study aimed to determine age- and sex-specific reference values for CT-based skeletal muscle markers in a healthy population, and to correlate them with serum creatinine and 24-h urinary creatinine excretion (24h-UCE).

**Methods:**

Skeletal muscle index (SMI) - a marker of muscle mass/quantity - and skeletal muscle radiation attenuation (SMRA) and intermuscular adipose tissue index (IMATI) - markers of muscle quality/myosteatosis - were determined using a deep-learning-based method from axial CT images at the level of the 3^rd^ lumbar vertebra in living kidney donors assessed between 01/2005 and 05/2023. Age- and sex-specific reference values were determined by the 5^th^ percentile, and correlation was tested with the Pearson correlation coefficient.

**Results:**

CT scans of 394 healthy individuals were included. The mean age was 53 years (SD 12), mean BMI was 25.2 kg/m^2^ (SD 3.9), and 130 patients (33%) were male. The reference values for low skeletal muscle mass (SMI) in males were 43.7 cm^2^/m^2^ (20–39 years), 44.9 cm^2^/m^2^ (40–59 years), and 39.7 cm^2^/m^2^ (≥60 years). In females, the corresponding values were 33.8 cm^2^/m^2^ (20–39 years), 34.8 cm^2^/m^2^ (40–59 years), and 31.2 cm^2^/m^2^ (≥60 years). SMI showed a moderate correlation with serum creatinine (r = 0.452, p < 0.001) but a weak correlation with 24h-UCE (r = 0.188, p = 0.003). Correlations were all weak for SMRA (creatinine: r = 0.220, p < 0.001; 24h-UCE: r = 0.177, p = 0.006) and IMATI (creatinine: r = −0.101, p = 0.054; 24h-UCE, r = −0.108, p = 0.093).

**Conclusion:**

The age- and sex-specific reference values reported here could be used in clinical practice and future studies to identify patients at risk of muscle decline.

## 1 Introduction

Muscle mass plays a crucial role in overall health and physical function, serving as a key determinant of strength and metabolic regulation (Janssen et al., 2002). The European Working Group on Sarcopenia in Older People (EWGSOP) defined sarcopenia as an involuntary loss of muscle mass occurring physiologically with aging and a qualitative alteration in muscle function and strength (Cruz-Jentoft et al., 2019). Skeletal muscle constitutes the largest organ in humans, representing approximately 40% of the total body weight (Pedersen and Febbraio, 2012). The prevalence of sarcopenia in healthy individuals increases with advanced age, ranging from 9% at 45 years and up to 64% in individuals aged over 85 years (Cherin et al., 2014). In terms of muscle quality, fatty infiltration (i.e., myosteatosis) has also been associated with poor clinical outcomes (Westenberg et al., 2023; Kim and Kim, 2021). Indeed, muscle quality, which is related to myosteatosis, is as important as muscle quantity in health and aging, and influences muscle strength (Kim and Kim, 2021). It has been shown that the decline of muscle strength was much more rapid than the concomitant loss of muscle mass (Goodpaster et al., 2006). However, muscle quality has been less explored compared with muscle mass, and a standard definition and its assessment methods have not been clearly established yet (Kim and Kim, 2021).

Different strategies to determine muscle mass and quality have been proposed, but the method of choice remains controversial. Creatinine, a metabolite derived from muscle creatine breakdown, is commonly used as an indirect indicator of muscle mass, given its stable production and renal excretion (Patel et al., 2013). Biologically, 24-hour urinary creatinine excretion rate (24h-UCE) is considered as a useful marker which is associated with muscularity (Hiller et al., 2023; Wang et al., 1996). The whole-body muscle area and density can also be predicted by computed tomography (CT) at the level of the 3^rd^ lumbar vertebra (Kong et al., 2022). This approach is recognized by the EWGSOP consensus (Cruz-Jentoft et al., 2019). It is possible to measure different body composition markers from a CT scan requested as part of an oncological or preoperative assessment before surgery for example. This imaging modality has proven to be an accurate and objective way for assessing muscle mass and quality, respectively, and with advancements in artificial intelligence (AI), its accuracy and speed are rapidly increasing (Westenberg et al., 2023). AI can help to automatically differentiate the proportion of various tissues, in particular by measuring muscle and adipose tissue indices, such as skeletal muscle area (SMA), skeletal muscle radiation attenuation (SMRA), and intermuscular adipose tissue (IMAT) area (Koitka et al., 2021). As muscle is dependent on age, sex, ethnicity, and BMI, large variation exists for cut-offs defining low muscle mass and quality. However, the cut-offs used in studies are usually not corrected for these factors, and could result in over- or underestimation of the muscle status.

The present study aimed to determine age- and sex-specific reference values for CT-based low skeletal muscle quantity and quality markers in a healthy population, and to correlate them with serum creatinine and 24h-UCE.

## 2 Methods

### 2.1 Patients

This retrospective single-center study included all consecutive patients who were considered and assessed for living donor nephrectomy at the Organ Transplant Center (CTO), Lausanne University Hospital (CHUV), Lausanne, Switzerland, between January 2005 and May 2023. All patients were managed according to a standardized protocol (psychological, ethical, medical, and surgical evaluation). Potential donors who were assessed for donation but did not undergo the nephrectomy for various reasons (revocation of consent to donation, donor-recipient incompatibility, discovery of an illness or pathology contraindicating donation) were included. Routine CT angiography was requested for all potential donors. Exclusion criteria comprised technically insufficient CT image quality (interfering artifacts, e.g. metal or motion), iodine allergy with replacement of CT by MRI, or patients who did not provide consent for reuse of their data. Patient demographics and biochemical data were collected. The estimated glomerular filtration rate (eGFR, mL/min/1.73 m^2^) was calculated using the CKD-EPI equation (Levey et al., 2006). Measurement of creatinine clearance was calculated by multiplying the ratio of urine creatinine to plasma creatinine by 24-hour urine volume, expressed in mL/min, and normalized to a body surface area of 1.73 m^2^ (Doolan et al., 1962).

### 2.2 Skeletal muscle mass and quality assessment

**FIGURE 1:**
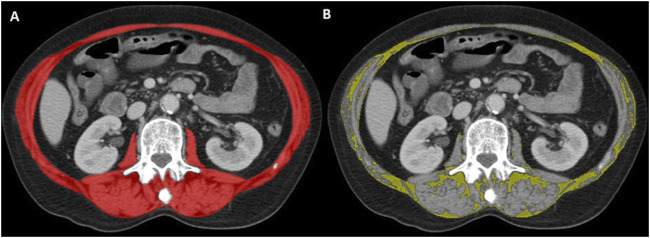
Representative axial CT image at the level of the 3^rd^ lumbar vertebra. Artificial intelligence-based segmentation of abdominal wall and paraspinal muscles in red **(A)**. This surface was used to determine the skeletal muscle area (SMA; red minus yellow surface, not shown) and intermuscular adipose tissue (IMAT) area in yellow **(B)** according to standard threshold values (−29 to 150 HU for muscle, −190 to −30 HU for fat).

Multiphase CT scans were performed after intravenous contrast medium administration. Axial CT images were extracted from the picture archiving and communication system (PACS) in the digital imaging and communications in medicine (DICOM) format, and included the psoas, paraspinal, and abdominal wall muscles at the level of the 3^rd^ lumbar vertebra (Figure 1). Muscle segmentations were obtained using a deep-learning-based method with a U-Net architecture algorithm, and secondarily reviewed and corrected as appropriate by a board-certified musculoskeletal radiologist (Koitka et al., 2021; Ibtehaz and Rahman, 2020). To assess muscle quantity/mass, the SMA was measured in cm^2^ and normalized by the square of patient height to obtain the skeletal muscle index (SMI) in cm^2^/m^2^. To evaluate muscle quality, the SMRA was measured in Hounsfield unit (HU). The IMAT area assessed the fat pixels located between muscles, and was also normalized by the square of patient height to obtain the IMAT index (IMATI, cm^2^/m^2^). Both SMRA and IMATI can be used as markers of myosteatosis (Westenberg et al., 2023).

### 2.3 Statistical analysis

Continuous data were presented as mean (standard deviation, SD) or median (interquartile range, IQR) and compared with Student’s t-test or Mann-Whitney U test, as appropriate. Categorical variables were presented as frequencies (%) and analyzed with Pearson’s chi-squared or Fisher’s exact test, as appropriate. Demographic and clinical data of male subjects were compared to female subjects. Living kidney donors were stratified by age (20–39, 40–59, ≥60 years) and body mass index (BMI; <25, 25–29.9, ≥30 kg/m^2^). Reference values for low SMA, SMI, SMRA, IMAT area, and IMATI were determined by the 5^th^ percentile (p5) and the mean value minus two SDs, as previously described (Westenberg et al., 2023; Kong et al., 2022; van der Werf et al., 2018). These reference values were stratified according to age, sex, and BMI categories. Statistical correlations between skeletal muscle markers and clinical data were measured using the Pearson correlation coefficient and interpreted as follows: 0.00–0.09 negligible; 0.10–0.39 weak; 0.40–0.69 moderate; and 0.70–0.89 strong correlation. A P-value ≤0.05 was considered statistically significant, and all analyses were performed using SPSS 29.0 software (SPSS Inc., Chicago, IL).

## 3 Results

A total of 394 healthy individuals (i.e., living kidney donors) were included. The mean age of the study population was 53 years (SD 12), mean BMI was 25.2 kg/m^2^ (SD 3.9), and 130 patients (33%) were males. Males had significantly higher BMI than female patients, but were comparable in terms of age and ethnicity (Table 1). They also had significantly higher mean SMA, SMI, and SMRA, while mean IMAT area and IMATI were comparable. SMI was 1.30-fold higher in males than in females. Biologically, serum creatinine (82.6 vs. 68.3 μmol/L, p < 0.001) and 24h-UCE (108.0 vs. 100.5 mL/min/1.73 m^2^, p < 0.001) were significantly higher in males than in female subjects.

**TABLE 1:** Baseline characteristics of the study population.

	Overall population (*n* = 394)		
	Male (n = 130)	Female (n = 264)	P-value
Age (years)	51.3 (SD 14.0)	53.3 (SD 11.4)	0.210
BMI (kg/m^2^)	25.5 (SD 5.0)	25.0 (SD 4.3)	0.037
Ethnicity			0.210
White	113 (87%)	242 (93%)	
Black/African	11 (8%)	9 (3%)	
Hispanic/Latino	2 (2%)	6 (2%)	
Asian	4 (3%)	7 (2%)	
Weight (kg)	78.7 (SD 11.5)	66.8 (SD 11.6)	**< 0.001**
Height (cm)	175.4 (SD 7.5)	163.2 (SD 6.1)	**< 0.001**
SMA (cm^2^)	163.6 (SD 26.6)	108.5 (SD 14.4)	**< 0.001**
SMI (cm^2^/m^2^)	53.1 (SD 7.6)	40.8 (SD 5.5)	**< 0.001**
SMRA (HU)	42.3 (SD 6.5)	37.8 (SD 6.8)	**< 0.001**
IMAT area (cm^2^)	12.8 (SD 6.8)	12.0 (SD 6.7)	0.259
IMATI (cm^2^/m^2^)	4.2 (SD 2.2)	4.5 (SD 2.5)	0.224
Serum creatinine (μmol/L)	82.6 (SD 11.6)	68.3 (SD 10.2)	**< 0.001**
eGFR (mL/min/1.73 m^2^)	92.5 (13.5)	89.1 (15.3)	0.071
24-h UCE (ml/min/1.73 m^2^)	108.0 (17.3)	100.5 (20.0)	**< 0.001**

BMI, body mass index; SMA, skeletal muscle area; SMI, skeletal muscle index; SMRA, skeletal muscle radiation attenuation; IMAT, intermuscular adipose tissue; IMATI, IMAT index. Significant p-values (<0.05) are displayed in bold characters.

The reference values (calculated as p5) for low skeletal muscle mass (SMI) in males were 43.7 cm^2^/m^2^ (20–39 years), 44.9 cm^2^/m^2^ (40–59 years), and 39.7 cm^2^/m^2^ (≥60 years) (Table 2). In females, reference values for SMI were 33.8 cm^2^/m^2^ (20–39 years), 34.8 cm^2^/m^2^ (40–59 years), and 31.2 cm^2^/m^2^ (≥60 years). The reference values for SMA, SMRA, IMAT area, and IMATI according to sex and BMI category are presented in Table 2. The reference values according to the mean values minus two SDs are presented in Supplementary Table S1.

**TABLE 2:** Reference values for SMI, SMA, and SMRA, per age and BMI category (5^th^ percentile).

Age, years	20–39		40–59		≥60	
	Male	Female	Male	Female	Male	Female
SMA, cm^2^						
All BMI’s	132.3	88.8	142.4	89.8	123.9	79.7
<25	125.5	86.7	119.5	80.3	117.2	79.7
25–29.9	158.5	^a^	144.9	96.7	131.2	88.9
≥30	^a^	112.1	160.8	104.0	^a^	101.0
SMI, cm^2^/m^2^						
All BMI’s	43.7	33.8	44.9	34.8	39.7	31.2
<25	43.7	32.5	40.7	32.9	38.2	31.2
25–29.9	46.3	^a^	47.9	36.6	43.7	32.8
≥30	^a^	42.1	51.7	35.1	^a^	35.5
SMRA, HU						
All BMI’s	42.1	35.0	32.2	28.4	27.7	25.2
<25	43.5	39.6	41.5	31.6	28.4	26.3
25–29.9	38.4	^a^	32.6	28.2	27.3	23.7
≥30	^a^	32.7	29.2	16.1	^a^	20.1
IMAT, cm^2^						
All BMI’s	4.7	3.5	4.8	3.8	7.1	7.0
<25	4.1	3.5	4.8	2.7	6.9	6.7
25–29.9	5.0	^a^	6.7	6.2	10.2	10.2
≥30	^a^	8.8	12.3	8.5	^a^	11.3
IMATI, cm^2^/m^2^						
All BMI’s	1.4	1.3	1.7	1.4	2.4	2.7
<25	1.3	1.3	1.4	1.1	2.3	2.6
25–29.9	1.6	^a^	2.0	2.2	3.8	3.8
≥30	^a^	3.4	4.1	3.5	^a^	4.6

^a^ Number of donors too small to perform analyses (at least ten).

SMA, skeletal muscle area; SMI, skeletal muscle index; SMRA, skeletal muscle radiation attenuation; IMAT, intermuscular adipose tissue; IMATI, IMAT index.

**FIGURE 2:**
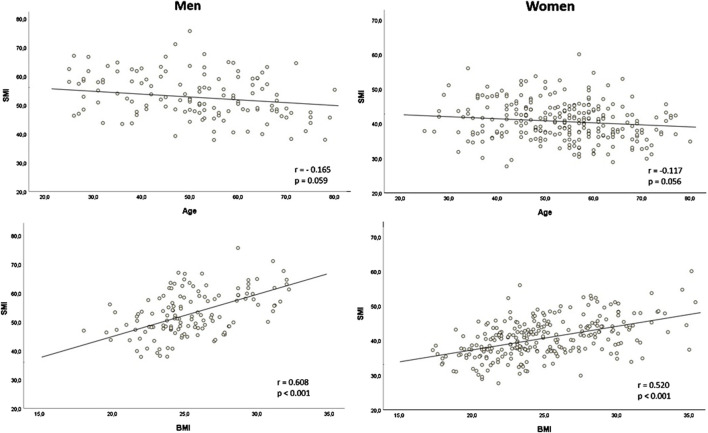
Correlation of skeletal muscle index (SMI) with age and body mass index (BMI) according to sex SMI in cm^2^/m^2^; BMI in kg/m^2^; r: Pearson’s r.

SMI showed a weak negative correlation with age in both males (r = −0.165, p = 0.059) and females (r = −0.117, p = 0.056, Figure 2). SMRA and IMATI also correlated moderately with age in males and females (Supplementary Figure S1). SMI had a moderate correlation with BMI in males (r = 0.608, p < 0.001) and females (r = 0.520, p < 0.001, Figure 2). The correlation between SMRA and BMI was negligible in males (r = −0.043, p = 0.627) and weakly negative in females (r = −0.223, p < 0.001), while the correlation between IMATI and BMI was both moderate in males (r = 0.548, p < 0.001) and females (r = 0.446, p < 0.001, Supplementary Figure S1). The two muscle quality markers–SMRA and IMATI–were strongly negatively correlated (r = −0.814, p < 0.001).

The correlation between SMI, serum creatinine, and 24h-UCE is presented in Supplementary Figure S2. SMI showed a moderate correlation with serum creatinine (r = 0.452, p < 0.001) but a weak correlation with 24h-UCE (r = 0.188, p = 0.003). Correlations were all weak between SMRA, serum creatinine (r = 0.220, p < 0.001), and 24h-UCE (r = 0.177, p = 0.006), and between IMATI, serum creatinine (r = −0.101, p = 0.054) and 24h-UCE (r = −0.108, p = 0.093).

## 4 Discussion

In the present study, age- and sex-specific reference values are reported for CT-based low skeletal muscle markers, including muscle quantity and quality (myosteatosis) in a healthy subject population. These cut-off values may help to improve clinical identification of patients at risk of muscle decline. In addition, radiological analysis of body composition markers using CT scans could make it possible to individualize treatment decisions and allow reconditioning strategies in most oncological and surgical populations, as CT scans are often performed routinely.

Determining the presence of low muscle mass and myosteatosis may help in assessing whether a patient is fit for invasive treatments, such as surgery or chemotherapy in cancer care, and could also potentially prevent their side effects by introducing intervention measures earlier (Shachar et al., 2016). Indeed, the population is changing, with older and morbid individuals requiring optimization of medical assessment, screening, and personalized strategies to preserve good treatment outcomes (Westenberg et al., 2023). Living kidney donors represent a unique cohort, as they are healthy subjects rather than patients (Abe et al., 2003). Few studies have described reference values for both skeletal muscle mass and quality measured using CT in a healthy population. Of these studies, only a few reported reference values for SMA, SMI, and SMRA stratified by age, sex, and BMI, and they were slightly different from the present results (Westenberg et al., 2023; van der Werf et al., 2018; Abe et al., 2003). These differences could be explained by variations in demographics, age categories, CT protocols and image acquisition parameters, type and amount of contrast medium, contrast timing, and the variation in automated measures and manual delineation at the level of the 3^rd^ lumbar vertebra (Westenberg et al., 2023). Furthermore, some differences exist in the methods of data analysis, such as 2 SDs below the mean, the 5^th^ percentile value, or even the 10^th^ or 25^th^ percentile. In the present study, the values are reported using two methods, and important differences according to age and BMI categories could also be observed.

In this study, male subjects were the same age and ethnicity as female subjects, but showed significantly higher BMIs. Furthermore, males also had higher mean SMA, SMI, and SMRA, while mean IMAT area and IMATI were comparable to females. It is widely reported that body composition differs between males and females, with men generally having more muscle mass (Abe et al., 2003). This is confirmed by the present results, as the SMA and SMI were significantly higher in male subjects across all age and BMI categories. This is comparable to previous retrospective studies in healthy Caucasian and Asian subjects, where the SMI was respectively 1.31- and 1.53-fold higher in males than in females (Kong et al., 2022; van der Werf et al., 2018). Interestingly, our results presented more discrepancy when it came to assessing muscle quality, to the extent that the SMRA was higher in men, but the IMATI was comparable. One retrospective study also showed that the SMRA was lower in women than in men (Anderson et al., 2013). Two longitudinal studies have shown that the IMATI increased according to age, which is in line with our results which suggested a significant moderate positive correlation (males: r = 0.491, p < 0.001; females: r = 0.500, p < 0.001) (Song et al., 2004; Delmonico et al., 2009). Interestingly, it has been described that in older adults, the degree of muscle strength loss was greater than that of muscle mass loss, and that aging was associated with an increase in IMATI regardless of changes in weight (Delmonico et al., 2009). Similarly, our results showed that the SMI did not vary with age, while the SMRA and IMATI were significantly correlated with the change in age.

The mean attenuation of muscles (SMRA) reflects their lipid content, such that a lower muscle attenuation reflects a higher lipid content (Goodpaster et al., 2000). This phenomenon has been observed in patients with comorbidities, such as obesity, type 2 diabetes, advanced age, and cancer (Aubrey et al., 2014). The present study confirmed that older age was significantly correlated with lower SMRA in both men and women, which is in line with other studies (Westenberg et al., 2023; Anderson et al., 2013). Interestingly, the SMRA did not correlate with the BMI, while the SMI and IMATI correlated positively with the BMI, meaning that the higher the BMI, the higher the SMI and IMATI. Other retrospective study showed similar correlations (Kong et al., 2022; van Vugt et al., 2019). Two important messages should be mentioned here: firstly, that BMI is not necessarily an adequate anthropometric measurement reflecting body composition, and secondly, that the two indices of muscle quality (SMRA and IMATI) represent different entities. The present study showed that these two parameters were negatively correlated: the more the SMRA decreased (muscle density), the more the IMATI (intermuscular fat) increased. One of the issues is that CT images can be influenced by contrast enhancement, and most of the clinical studies did not disclose the use of contrast medium. Therefore, the average muscle density and intermuscular surface have some limitations for the comparison of data among different institutions using various CT protocols (Kim and Kim, 2021). Furthermore, the IMATI is a muscle quality index that includes the visible storage of lipids in adipocytes located between muscle groups (intermuscular fat). In a retrospective study of 20’664 healthy subjects, the proportion of IMATI in men and women was respectively only 3.3% and 4.8% of total trunk muscle area (Kim et al., 2021). Thus, the IMATI could lead to an underestimation of the differences in myosteatosis between individuals (Kim and Kim, 2021). Another study highlighted that the accumulation of intramuscular fat was linked to metabolic dysfunction, including impaired glucose tolerance, which may be relevant in the context of prediabetes (Lopez-Pedrosa et al., 2024). Other metabolic parameters, such as cholesterol levels or HbA1c, should be assessed in the future.

In this study, SMI showed a moderate positive correlation with serum creatinine, thus confirming its inherent relation to muscle mass. This supports that CT imaging is an accurate method for analysis of skeletal muscle, but if not available, serum creatinine could be an alternative. However, SMI was only poorly correlated with 24h-UCE. Males had higher serum creatinine and 24h-UCE compared to females, which is similar to a previous retrospective on living kidney donors (Westenberg et al., 2023). It has also been shown that serum creatinine levels decreased more significantly in older males than in older females due to sex-specific muscle mass decline (Yim et al., 2023). On the contrary, a retrospective study of 2’974 subjects showed that measured GFR declined after 35 years of age, and that the linear decline was faster in females compared to males (7.7 vs. 6.6 mL/min/1.73 m^2^/decade, p = 0.013) (Fenton et al., 2018). The poor correlation between muscle SMI and 24h-ECU may be partly attributed to errors in urine collection, making the results less reliable and difficult to compare. Additionally, creatinine excretion is influenced by the patient’s hydration status, diet, and other metabolic factors (Wulkan and van der Horst, 2021).

This study has several limitations that need to be acknowledged. The main limitation is its retrospective nature and limited sample size. There may also be several sources of variability in the measurement of skeletal muscle mass and quality markers. In addition, the impact of different human observers with varying levels of expertise validating/correcting the AI-based automated segmentations was not assessed. Different anatomical measurement sites may yield different muscle mass estimates. While several studies have explored the relevance of the psoas and thigh muscle indices, the present study focused on muscles at the 3^rd^ lumbar vertebral level to the extent that it is recognized by the latest EWGSOP and that our center developed expertise for this measurement (Cruz-Jentoft et al., 2019; Oka et al., 2025; Coban et al., 2024). Even if it was a healthy population *a priori* since evaluated for living kidney donation, there were also several confounding factors (e.g., nutritional status, comorbidities) that could have had an impact on body composition markers and that were not taken into consideration during the analysis. No metabolic markers, such as cholesterol levels or HbA1c, were included in this study, and the relationship between muscle lipid deposition and these parameters could not be assessed. Renal function and the distribution of the contrast medium could also cause bias in the measurement of skeletal muscle indices on CT. Ethnic origin can also play a role on body composition, but further analyses were not carried out given the preponderance of Caucasians. The thresholds presented here should be further investigated prospectively, as they were not analyzed for clinical outcomes.

In conclusion, this study provides reference values for CT-based low skeletal muscle quantity/mass and quality/myosteatosis stratified by age, sex, and BMI in healthy subjects. These values could be used in clinical practice and future studies to identify patients at risk of muscle decline who could benefit from interventional measures.

## Data Availability

The raw data supporting the conclusions of this article will be made available by the authors, without undue reservation.
