# Supplementary material for: Age- and sex-specific reference values for CT-based low skeletal muscle quantity and quality in healthy living kidney donors
Source: Front Physiol. 2025 Apr 25;16:1566463. doi: 10.3389/fphys.2025.1566463 (PMC12061965; doi:10.3389/fphys.2025.1566463)
Supplement: Supplementary file 1 [file Table1.docx]

**Table S1** Reference values for SMI, SMA, and SMRA, per age and BMI category (Mean – 2SD)

| **Age, years** | **20–39** | | **40–59** | | **≥ 60** | |
| --- | --- | --- | --- | --- | --- | --- |
|  | **Male** | **Female** | **Male** | **Female** | **Male** | **Female** |
| **SMA, cm^2^** | | | | | | |
| **All BMI’s** | 125.7 | 83.7 | 118.8 | 83.11 | 107.2 | 68.7 |
| <25 | 116.2 | 81.0 | 119.6 | 78.8 | 109.5 | 76.6 |
| 25-29.9 | 154.4 | * | 117.0 | 90.04 | 116.0 | 78.5 |
| ≥30 | * | 109.5 | 154.5 | 98.8 | * | 88.7 |
| **SMI, cm^2^/m^2^** | | | | | | |
| **All BMI’s** | 41.3 | 29.6 | 39.2 | 31.80 | 36.4 | 25.5 |
| <25 | 39.30 | 28.8 | 39.0 | 30.7 | 36.5 | 28.9 |
| 25-29.9 | 45.5 | * | 40.0 | 34.2 | 39.2 | 29.0 |
| ≥30 | * | 42.1 | 46.8 | 35.8 | * | 32.8 |
| **SMRA, HU** | | | | | | |
| **All BMI’s** | 40.2 | 35.0 | 30.2 | 26.4 | 26.8 | 18.8 |
| <25 | 41.8 | 36.6 | 37.8 | 30.3 | 28.8 | 23.4 |
| 25-29.9 | 36.7 | * | 29.9 | 25.3 | 24.9 | 20.3 |
| ≥30 | * | 32.1 | 29.2 | 20.1 | * | 18.1 |
| **IMAT, cm^2^** | | | | | | |
| **All BMI’s** | 0.1 | 0.42 | -3.0 | -0.3 | 4.6 | 1.0 |
| <25 | 1.7 | 2.0 | 1.5 | 1.0 | 4.0 | 2.8 |
| 25-29.9 | 0.1 | * | -0.9 | 1.1 | 8.1 | 6.0 |
| ≥30 | * | 5.8 | 12.3 | 4.0 | * | 1.7 |
| **IMATI, cm^2^/m^2^** | | | | | | |
| **All BMI’s** | 0.1 | -0.1 | -0.1 | -0.1 | 1.8 | 0.6 |
| <25 | 0.5 | 0.8 | 0.7 | 0.4 | 1.4 | 0.9 |
| 25-29.9 | 0.1 | * | -0.3 | 0.3 | 3.1 | 2.4 |
| ≥30 | * | 1.7 | 4.1 | 1.9 | * | 0.1 |

* Number of donors too small to perform analyses (at least ten).

SMA: skeletal muscle area; SMI: skeletal muscle index; SMRA: skeletal muscle radiation attenuation; IMAT: intermuscular adipose tissue; IMATI: IMAT index
