# Supplementary figures and images for: Age- and sex-specific reference values for CT-based low skeletal muscle quantity and quality in healthy living kidney donors
Source: Front Physiol. 2025 Apr 25;16:1566463. doi: 10.3389/fphys.2025.1566463 (PMC12061965; doi:10.3389/fphys.2025.1566463)

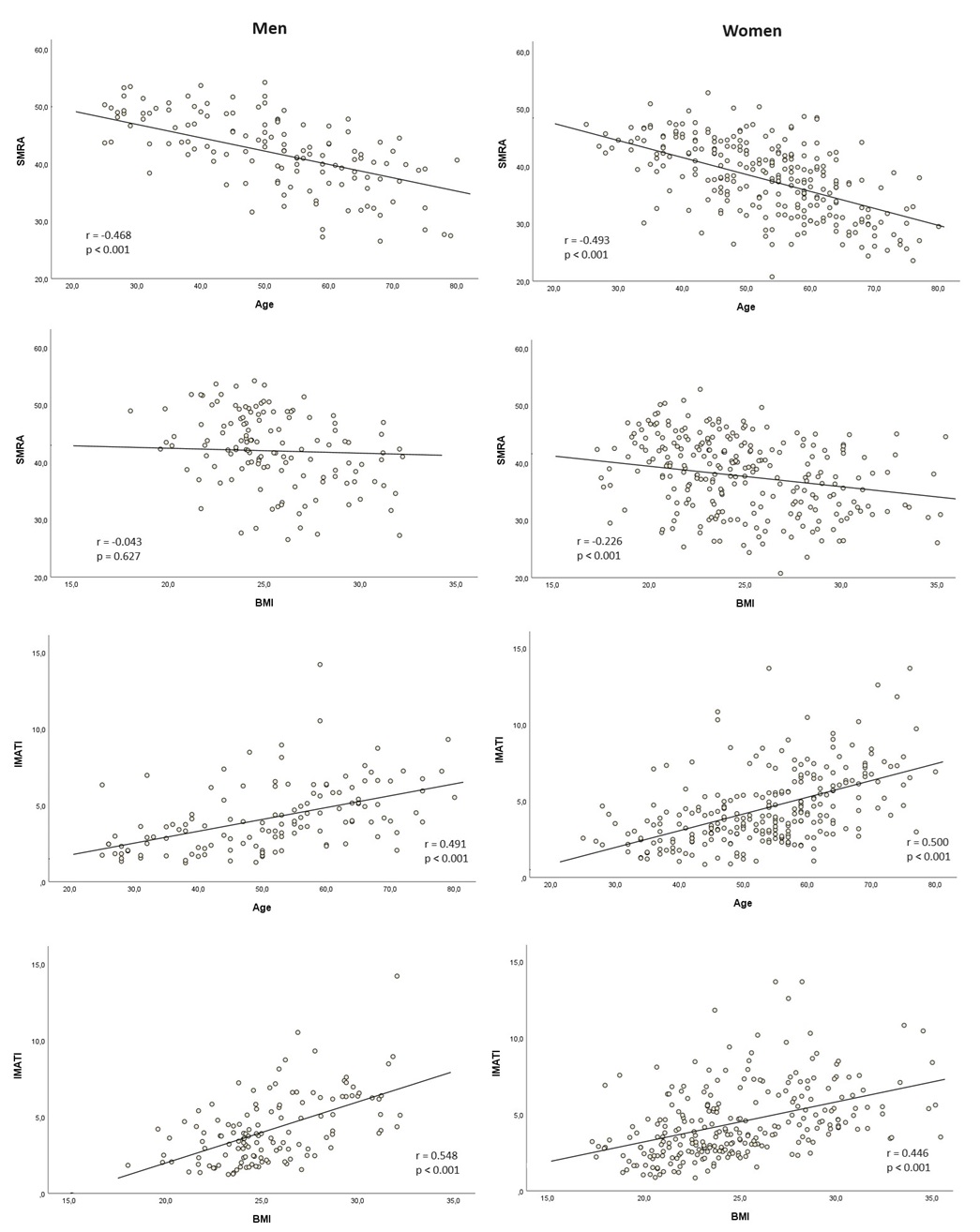

Supplement: Supplementary file 2 [file Image1.jpeg]

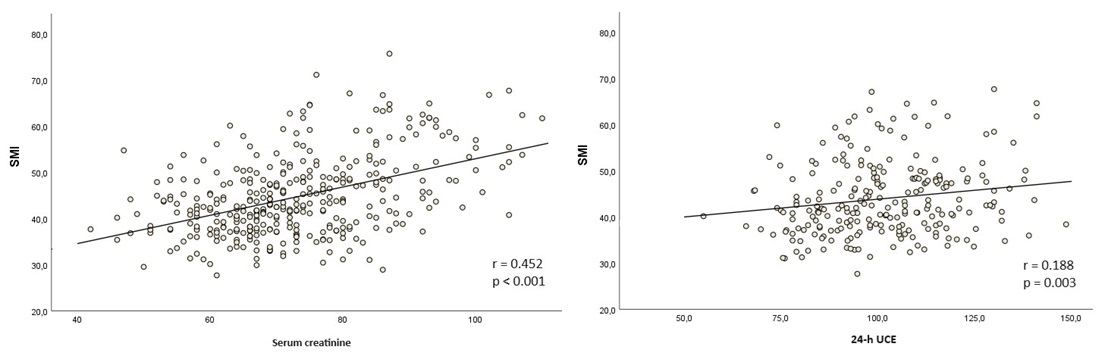

Supplement: Supplementary file 3 [file Image2.jpeg]
